# Leucine-rich alpha-2 glycoprotein as a marker of mucosal healing in inflammatory bowel disease

**DOI:** 10.1038/s41598-021-90441-x

**Published:** 2021-05-27

**Authors:** Eriko Yasutomi, Toshihiro Inokuchi, Sakiko Hiraoka, Kensuke Takei, Shoko Igawa, Shumpei Yamamoto, Masayasu Ohmori, Shohei Oka, Yasushi Yamasaki, Hideaki Kinugasa, Masahiro Takahara, Keita Harada, Masaki Furukawa, Kouichi Itoshima, Ken Okada, Fumio Otsuka, Takehiro Tanaka, Toshiharu Mitsuhashi, Jun Kato, Hiroyuki Okada

**Affiliations:** 1 Department of Gastroenterology and Hepatology, Okayama University Graduate School of Medicine, Dentistry and Pharmaceutical Sciences, 2-5-1 Shikata-cho, Kita-ku, Okayama, 700-8558 Japan; 2 Department of Laboratory Medicine, Okayama University Hospital, Okayama, Japan; 3 Department of General Medicine, Okayama University Graduate School of Medicine, Dentistry and Pharmaceutical Sciences, Okayama, Japan; 4 Department of Pathology, Okayama University Graduate School of Medicine, Dentistry and Pharmaceutical Sciences, Okayama, Japan; 5 Center for Innovative Clinical Medicine, Okayama University Hospital, Okayama, Japan; 6 Department of Gastroenterology, Graduate School of Medicine, Chiba University, Chiba, Japan

**Keywords:** Biomarkers, Gastroenterology

## Abstract

Leucine-rich alpha-2 glycoprotein (LRG) may be a novel serum biomarker for patients with inflammatory bowel disease. The association of LRG with the endoscopic activity and predictability of mucosal healing (MH) was determined and compared with those of C-reactive protein (CRP) and fecal markers (fecal immunochemical test [FIT] and fecal calprotectin [Fcal]) in 166 ulcerative colitis (UC) and 56 Crohn’s disease (CD) patients. In UC, LRG was correlated with the endoscopic activity and could predict MH, but the performance was not superior to that of fecal markers (areas under the curve [AUCs] for predicting MH: LRG: 0.61, CRP: 0.59, FIT: 0.75, and Fcal: 0.72). In CD, the performance of LRG was equivalent to that of CRP and Fcal (AUCs for predicting MH: LRG: 0.82, CRP: 0.82, FIT: 0.70, and Fcal: 0.88). LRG was able to discriminate patients with MH from those with endoscopic activity among UC and CD patients with normal CRP levels. LRG was associated with endoscopic activity and could predict MH in both UC and CD patients. It may be particularly useful in CD.

## Introduction

Inflammatory bowel disease (IBD) is a chronic idiopathic intestinal disorder that includes two forms of conditions: ulcerative colitis (UC) and Crohn’s disease (CD). Several etiologies of both these diseases have been found, but a complete cure has remained difficult to achieve. Therefore, continuous treatment accompanied by the appropriate assessment of the disease activity is necessary.

Imaging tests, particularly endoscopy, are the gold standard for assessing the disease activity, but it is important to use non-invasive and repeatable biomarkers in daily clinical practice. In particular, predicting mucosal healing (MH) with biomarkers is a mandatory clinical procedure, as the absence of symptoms does not always indicate MH^1,2^.

There have been many biomarkers developed that reflect the disease activity in patients with IBD. C-reactive protein (CRP) was the first acute-phase protein to be described and is an exquisitely sensitive systemic marker of inflammation and tissue damage. CRP is produced in the liver, and stimulation of the cytokine interleukin (IL)-6 is essential for inducing its expression^3^. This serum marker has often been used to assess the IBD activity but sometimes fails to reflect the disease activity, given that the pathogenesis of these diseases are not always dependent on IL-6. CRP has been reported to predict MH with a sensitivity of 0.77–0.82 and specificity of 0.32–0.40 for UC^4,5^, and a sensitivity of 0.83–0.92 and specificity of 0.70–0.89 for CD^5,6^. However, the low cut-off values (0.08–0.5 mg/dL for UC and 0.03–0.7 mg/dL for CD)^4–10^ may be inconvenient for use in clinical practice.

Fecal markers have frequently been used for patients with IBD. Fecal calprotectin (Fcal) in particular has abundant evidence regarding its correlation with the disease activity of IBD. Previous reports have demonstrated a sensitivity of 0.54–1.00 and specificity of 0.67–0.90 for the prediction of MH in UC^4,11–15^ and a sensitivity of 0.42–0.96 and specificity of 0.71–0.83 for the prediction of MH in CD with ileal disease^6,12,14,16,17^. The fecal immunological test (FIT) have shown an equivalent ability to Fcal for predicting MH in UC (sensitivity of 0.92–0.94 and specificity of 0.62–0.79)^11–15,18^, although the specificity for predicting MH in CD was relatively low (sensitivity of 0.88–0.96 and specificity of 0.36–0.48)^12,16^.

Leucine-rich alpha-2 glycoprotein (LRG) is a novel serum biomarker for various diseases found by using a proteomics approach in patients with rheumatoid arthritis^19^. Serum LRG was reported to be elevated in various autoimmune diseases with levels correlating to the disease activity in rheumatoid arthritis, systemic lupus erythematosus, adult-onset Still’s disease, systemic juvenile idiopathic arthritis, primary biliary cholangitis, and IBD^19–26^. The upregulation of LRG is associated with not only IL-6 but also IL-1β, TNFα, IL-22, etc., and the protein is produced in inflamed organs as well as in the liver^27,28^. Previous studies have reported that levels of serum LRG were better correlated with the disease activity of UC than CRP^21,23^.

However, no previous studies have described a detailed sensitivity analysis of LRG for predicting MH in UC and drew no comparison between LRG and fecal markers in the prediction of MH and the endoscopic activity. In addition, no reports have described the correlation between LRG and the CD activity.

Therefore, in the present study, we investigated the association of LRG with the endoscopic activity of IBD and the predictability of the marker for MH compared with the performance of CRP and fecal markers.

## Materials and methods

### Patients

All IBD patients who underwent colonoscopy or balloon-assisted enteroscopy (BAE) at Okayama University Hospital between November 2015 and November 2019 with serum and stool samples obtained on the day endoscopy were considered eligible. All UC patients and CD patients with colonic disease alone underwent colonoscopy, while CD patients who had lesions in the small bowel received BAE. All patients provided blood samples for the determination of serum LRG and CRP on the day of endoscopy. In addition, all patients were asked to collect, at home, two stool samples within 2 days before endoscopy and bring them to the hospital on the day of endoscopy for the determination of FIT and Fcal. The clinical characteristics of the patients, including the age at the diagnosis, sex, disease location, and current medications, were also recorded. The analyses were confined to a single endoscopy per patient. For patients with more than one endoscopy during the study period, the data of the first endoscopy was used.

The exclusion criteria were insufficient stool collection, having had a colostomy or ileostomy and failure to achieve full endoscopic observation for the patient’s lesions. Patients with other diseases that could affect the levels of LRG and CRP, including extraintestinal complications, collagen disease, heart failure, primary biliary cholangitis, infectious disease, and malignancy, at the time of endoscopy were excluded.

The clinical disease activity for UC patients was evaluated using the Mayo subscores for stool frequency (0, normal number for this patient; 1, 1–2 stools more than normal; 2, 3–4 stools more than normal; and 3, ≥ 5 stools more than normal) and rectal bleeding (0, no blood seen; 1, streaks of blood with stool less than half the time; 2, obvious blood with stool most of the time; and 3, blood alone passes)^29^. Clinical remission was defined as a Mayo stool frequency subscore of 0 or 1 and a Mayo rectal bleeding subscore of 0^30^. The clinical disease activity for CD patients was evaluated using the Crohn’s disease activity index (CDAI), with clinical remission defined as a CDAI < 150.

### Endoscopy procedures and the assessment of the endoscopic disease activity

Bowel preparation was performed with a polyethylene glycol-based or magnesium citrate-based electrolyte solution according to the standard protocol of our hospital. After the colonic lavage fluid had been cleared, the patients underwent colonoscopy or BAE. All BAE examinations were performed via the transanal route.

The endoscopic status of the UC patients was assessed according to the Mayo Endoscopic Subscore (MES) classification^29^. The MES is a four-point scale (0–3). The evaluation of MES was performed at each portion of the colorectum (cecum and ascending colon combined, transverse colon, descending colon, sigmoid colon, and rectum), and the maximum score in the colorectum of each patient was used for the analysis. Complete MH was defined as MES of 0, and MH was defined as MES of 0 or 1 throughout the colorectum. In addition, active inflammation was defined as MES 2 or 3.

For CD patients, the endoscopic assessment was scored according to the modified simple endoscopic score for Crohn’s disease (mSES-CD), which was modified for the evaluation of small bowel lesions as well as colonic lesions based on the original SES-CD^31^. The small intestine was divided into two segments: distal and proximal ileum. The distal ileum was defined as the portion of the ileum within 40 cm from the ileocecal valve or anastomosis, whereas the proximal ileum was defined as the deeper part of the ileum ≥ 40 cm proximal from those points^32^ mSES-CD was calculated by summing the scores of six bowel segments (proximal and distal parts of small intestine, right colon, transverse colon, left colon, and rectum). We excluded the scoring item “strictures” from the original SES-CD because it represents bowel damage rather than active inflammation. Thus, we evaluated the scores for the size of ulcers, ulcerated surface, and affected surface in each segment^6,33^. In addition, regarding anastomotic lesions, only ulcers (size score > 2) were defined as being derived from CD. Complete MH was defined as mSES-CD of 0, MH was defined as mSES-CD of 0–2, and active inflammation was defined as mSED-CD > 6. For additional analysis, mSES-CD including the strictures score was also used.

All endoscopic examinations were performed by experienced endoscopists, who scored the endoscopic findings with the results of the serum and fecal markers blinded.

### The measurement of serum LRG levels

The serum samples were collected by centrifuging blood samples at 1500 rpm for 15 min, dividing them into aliquots, and storing them frozen at − 80 °C until use. The serum LRG levels were analyzed in the in-hospital laboratory using NANOPIA LRG (SEKISUI MEDICAL Company Limited, Tokyo, Japan). The measuring regents were provided by Laboratory for SEKISUI MEDICAL Company Limited. The Laboratory for Sekisui Medical covered the cost of the regent for measuring LRG.

### The FIT analysis

The details of the method used for the FIT analysis have been described previously^11,18^. In brief, the patients prepared fecal samples using a Hemodia sampling probe (Eike Chemical, Tokyo, Japan). The submitted stool samples were immediately processed and examined using an OC-Sensor DIANA (Eiken Chemical) system, which can accurately measure fecal hemoglobin at concentrations of 50 to 1000 ng/mL. Fecal specimens with a hemoglobin concentration of > 1000 ng/mL were measured following dilution. Because FIT is not accurate for measuring hemoglobin concentrations of < 50 ng/mL, the specimens with a hemoglobin concentration within this range (0–50 ng/mL) were handled as category.

### The Fcal analysis

The fecal samples collected by the patients were stored at − 30 °C until shipment to the laboratory, where a calprotectin analysis was performed. The samples were sent to BML (Tokyo, Japan), where the level of calprotectin in the stool specimens was measured with a fluorescence enzyme immunoassay using Phadia EliA™ Calprotectin 2 (Thermo Fisher Scientific, Phadia AB, Uppsala, Sweden).

### Pathologic findings

In UC cases, histologic studies were evaluated using the Geboes score^34^ by a certificated pathologist. According to the scoring system, histologic disease activity of UC was classified into 6 grades from grade 0 to grade 5. For cases with endoscopic activity, the biopsy specimen from the site with maximum endoscopic activity was evaluated, while for remission cases, the biopsy specimen from the rectum was analyzed. Histological remission was defined as < Geboes score 2.1.

### Statistical analyses

Statistical analyses were conducted using the JMP software program, version 14.0 for Windows (SAS Institute Inc., Cary, NC, USA) and Stata/MP4, version 16.1 (Stata Corp. College Station, TX, USA). Spearman’s rank correlation test was performed to determine the correlation coefficient between the levels of the serum/fecal markers. The trend between the serum/fecal markers and the endoscopic scores was evaluated using the Jonckheere–Terpstra test. A receiver operating characteristics curve (ROC) analysis was used to assess the discriminatory performance of FIT, Fcal, LRG, and CRP for detecting the endoscopic status. The results were expressed as the area under the curve (AUC) with sensitivity, specificity, positive predictive value (PPV), and negative predictive value (NPV) with 95% confidence intervals (CIs). The cut off levels of LRG for complete MH using ROC analysis were ‘12.7 µg/mL’ for MES 0 in UC patients and ‘13.7 µg/mL’ for mSES-CD 0 in CD patients. Hence, the cutoff value of LRG was determined for prediction of complete MH in this study (13 µg/mL) for convenience in clinical use of both UC and CD. The cutoff values of the other biomarkers were set based on previous reports (CRP: 0.20 mg/dL, FIT: 100 ng/mL, and Fcal: 200 µg/g)^4–18,23^. In addition, AUC values for complete MH were compared with the statistical method described in the reference^35^. All *p*-values were two-sided. *p*-values of < 0.05 were considered to indicate statistical significance. The data underlying this article will be shared on reasonable request to the corresponding author.

### Ethical considerations

This study was approved by the Institutional Review Board of Okayama University Graduate School of Medicine (IRB number: 1904-035) conducted in accordance with the Declaration of Helsinki. All research was performed in accordance with relevant guidelines/regulations. Informed consent was obtained from each patient and/or their legal guardians.

## Results

### Clinical characteristics of the patients

A total of 222 endoscopies that were accompanied by corresponding biomarkers results were performed in 166 UC patients (79 men and 87 women; median age at the UC diagnosis, 33 years) and 56 CD patients (34 men and 22 women; median age at the diagnosis, 23 years).

The clinical characteristics and values of biomarkers of analyzed patients are summarized in Table 1. For UC patients, among 166 cases, 142 (86%) were in clinical remission, while the other 24 (14%) had clinically active disease. The colonoscopy findings were MES 0 in 77 (46%) cases, MES 1 in 57 (34%) cases, MES 2 in 25 (15%) cases, and MES 3 in 7 (4%) cases. The median (interquartile range [IQR]) value of the biomarkers was 11.6 (9.7–14.5) μg/mL for LRG, 0.06 (0.03–0.14) mg/dL for CRP, 50 (50–170) ng/mL for FIT, and 114 (35.1–283) μg/g for Fcal.

**Table 1:** Characteristics of the study patients, colonoscopy findings and results of serum/fecal markers.

	UC	CD
**Patients**		
Total	n = 166 (%)	n = 56 (%)
Gender		
Male/Female	79 (48)/87 (52)	34 (61)/22 (39)
Median (IQR) age at diagnosis (years)	33 (22–44)	23 (18–35)
Number of endoscopy procedures		
1/2/> 3	133 (80)/26 (16)/7 (4)	51 (91)/5 (9)/0 (0)
UC disease location		
Pancolitis/left-sided/proctitis	105 (63)/47 (28)/14 (8)	
CD disease location		
L1: ileal/L2: colonic/L3: ileocolonic		14 (25)/14 (25)/28 (50)
CD disease behavior		
B1: inflammation/B2: structuring/B3: penetrating		24 (43)/20 (36)/12 (21)
Perianal disease		17 (30)
Previous intestinal resection		25 (45)
**Endoscopy**		
Total colonoscopy/Balloon-assisted enteroscopy	166(100)/0 (0)	26 (46)/30 (54)
Median (IQR) duration of disease (months)	125 (51–213)	113 (45–222)
Median (IQR) age of undergoing endoscopy (years)	44 (34–57)	40 (28–47)
Clinical activity*		
Remission stage/Active stage	142 (86)/24 (14)	43 (77)/13 (23)
Indication for endoscopy		
Evaluation of disease		47 (84)
Surveillance		3 (5)
Stricture dilation		6 (11)
Concomitant medications		
5-aminosalycylic acid	147 (88)	49 (88)
Corticosteroids	9 (5)	3 (5)
Thiopurine	72 (43)	22 (39)
Tacrolimus	6 (4)	0 (0)
TNFα antagonist	21 (13)	33 (59)
Vedolizumab	3 (2)	0 (0)
Ustekinumab	0 (0)	3 (5)
Elemental diet		26 (46)
Endoscopy findings		
MES 0/1/2/3	77(46)/57(34)/25(15)/7(4)	
mSES-CD 0/1–2/3–4/5–10/11–15		19(34)/7(13)/9(16)/15(27)/6(11)
Histological findings**		
Geboes score 0/1/2/3/4/5	31(20)/41(26)/11(7)/26(16)/29(18)/20(13)	
Values of biomarkers, median (IQR)		
Leucin rich glycoprotein (µg/mL)	11.6 (9.7–14.5)	14.1 (9.6–17.8)
C-reactive protein (mg/dL)	0.06 (0.03–0.14)	0.12 (0.04–0.28)
Fecal immunochemical test (ng/mL)	50 (50–170)	50 (50–210)
Fecal calprotectin (μg/g)	114 (35.1–283)	196 (76.8–837)

*UC* ulcerative colitis, *CD* Crohn’s disease, *IQR* interquartile range, *MES* Mayo endoscopic subscore, *mSES-CD* modified simple endoscopic score for Crohn’s disease.

*Clinical remission was defined as a Mayo stool frequency subscore of 0 or 1 and a Mayo rectal bleeding subscore of 0 for UC, and Crohn’s disease activity index (CDAI) < 150 for CD.

**Of the total 166 UC cases, 158 underwent biopsy for pathological examinations.

For CD patients, among 56 endoscopy cases, 43 (77%) were in clinical remission, while the other 13 (23%) had clinically active disease. The endoscopy findings were mSES-CD 0 in 19 (34%) cases, mSES-CD 1–2 in 7 (13%) cases, mSES-CD 3–4 in 9 (16%) cases, mSES-CD 5–10 in 15 (27%) cases, and mSES-CD 11–15 in 6 (11%) cases. The median (IQR) value of biomarkers was 14.1 (9.6–17.8) μg/mL for LRG, 0.12 (0.04–0.28) mg/dL for CRP, 50 (50–210) ng/mL for FIT, and 196 (76.8–837) μg/g for Fcal.

### Correlation between LRG and other biomarkers in IBD patients

**Figure 1:**
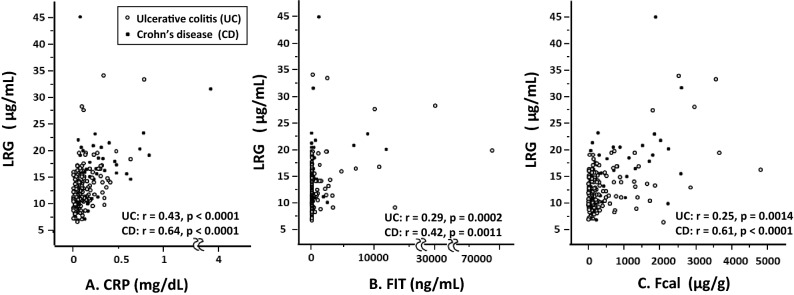
Correlation between LRG and the other serum/fecal biomarkers in IBD patients. (**A**) CRP, (**B**) FIT, (**C**) Fcal. *LRG* leucine-rich alpha-2 glycoprotein, *CRP* C-reactive protein, *FIT* fecal immunochemical test, *Fcal* fecal calprotectin. The round dots indicate the values of UC patients and the star dots indicate the values of CD patients.

The correlations between LRG and other biomarkers (CRP, FIT, and Fcal) were analyzed The Spearman’s rank correlation coefficients for UC and CD were as follows; CRP (Fig. 1A) UC:* r* = 0.43, *p* < 0.0001, CD:* r* = 0.64, *p* < 0.0001, FIT (Fig. 1B) UC*: r* = 0.29, *p* = 0.0002, CD: *r* = 0.42, *p* = 0.0011, and Fcal (Fig. 1C) UC: *r* = 0.25*, p* = 0.0014, CD: *r* = 0.61, *p* < 0.0001. In particular, the correlation coefficient between CRP and LRG was higher than those between fecal markers and LRG.

### Correlations between serum/fecal biomarkers and colonoscopic findings in UC patients

**Figure 2:**
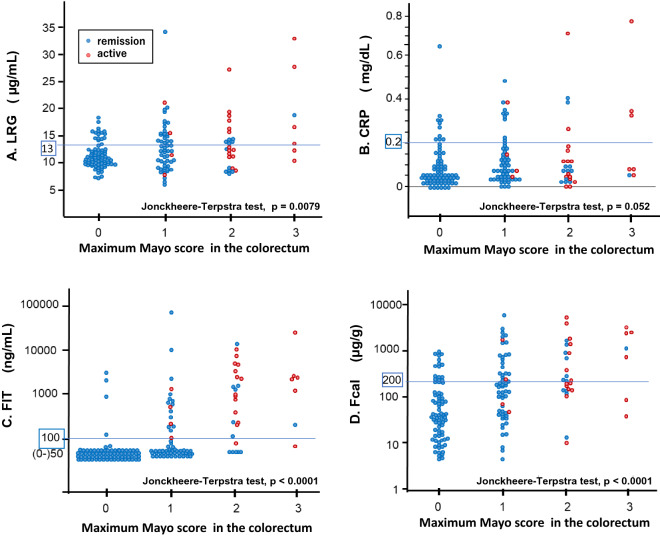
Correlation between the serum/fecal biomarkers and maximum MES in UC patients. (**A**) LRG, (**B**) CRP, (**C**) FIT, (**D**) Fcal. *MES* mayo endoscopic subscore, *UC* ulcerative colitis, *LRG* leucine-rich alpha-2 glycoprotein, *CRP* C-reactive protein, *FIT* fecal immunochemical test, *Fcal* fecal calprotectin. The red dots indicate the values of patients with active stage and the blue dots indicate the values of patients with remission stage. The specimens with a hemoglobin concentration within this range (0–50 ng/mL) were handled as category, because FIT is not accurate for measuring hemoglobin concentrations of < 50 ng/mL. Parallel blue lines showed the cutoff values of serum/fecal biomarkers in this study (LRG: 13.0 µg/mL, CRP: 0.20 mg/dL, FIT: 100 ng/mL, and Fcal: 200 µg/g).

The correlations between serum/fecal biomarkers and colonoscopic findings (maximum MES in the colorectum) were analyzed. The trend between the serum/fecal markers and the MES was statistically significant except for CRP (Jonckheere-Terpstra test: LRG: *p* = 0.0079, CRP: *p* = 0.052, FIT: *p* < 0.0001, and Fcal: *p* < 0.0001) (Fig. 2).

The sensitivity, specificity, PPV, NPV, and accuracy of serum/fecal markers in relation to MH were calculated for the 166 colonoscopy cases. The AUC for complete MH (MES 0) were LRG: 0.61, CRP: 0.59, FIT: 0.75, and Fcal: 0.72, respectively. The sensitivity and specificity for complete MH were LRG: 0.78 and 0.47, CRP: 0.87 and 0.18, FIT: 0.95 and 0.47, and Fcal: 0.77 and 0.47, respectively (Table 2). The AUC value of LRG was significantly lower than those of the two fecal markers (LRG vs. FIT, *p* = 0.0038, and LRG vs. Fcal, *p* = 0.039). Sensitivity analysis for active inflammation (MES 2 or 3) are shown in Supplemental Table S1.

**Table 2:** Sensitivity, specificity, and predictive values of the serum/fecal biomarkers for MES 0 in UC patients.

	AUC	Sensitivity	Specificity	PPV	NPV	Accuracy
LRG	0.61	0.78 (0.69–0.87)	0.47 (0.37–0.58)	0.56 (0.47–0.65)	0.71 (0.60–0.83)	0.61 (0.54–0.69)
CRP	0.59	0.87 (0.80–0.95)	0.18 (0.10–0.26)	0.48 (0.40–0.56)	0.62 (0.43–0.80)	0.50 (0.42–0.58)
FIT	0.75*	0.95 (0.90–1.00)	0.47 (0.37–0.58)	0.61 (0.52–0.70)	0.91 (0.83–0.99)	0.69 (0.62–0.76)
Fcal	0.72*	0.77 (0.67–0.86)	0.47 (0.37–0.58)	0.56 (0.46–0.65)	0.70 (0.58–0.82)	0.61 (0.53–0.68)

*MES* Mayo endoscopic subscore, *UC* ulcerative colitis, *AUC* area under curve, *PPV* positive predictive value, *NPV* negative predictive value, *LRG* leucine-rich alpha-2 glycoprotein, *FIT* fecal immunochemical test, *Fcal* fecal calprotectin, *CRP* C-reactive protein.

*The AUC value of LRG was significantly lower than those of the two fecal markers (LRG vs. FIT, *p* = 0.0038, LRG vs. Fcal, *p* = 0.039).

We analyzed the association of serum/fecal markers with complete MH (MES 0) in UC patients with normal serum CRP levels (≤ 0.2 mg/dL). The LRG, FIT, and Fcal, levels were associated with complete MH (MES 0) in 140 patients with normal serum CRP levels (MES 0 vs. MES 1–3: LRG *p* = 0.018, FIT *p* < 0.0001, and Fcal *p* < 0.0001) (Supplemental Fig. S1). The AUC for complete MH (MES 0) were LRG: 0.62, FIT: 0.72, and Fcal: 0.70, respectively. The sensitivity and specificity for complete MH (MES 0) were LRG: 0.87 and 0.41, FIT: 0.94 and 0.42, and Fcal: 0.76 and 0.41, respectively (Table 3).

**Table 3:** Sensitivity, specificity, and predictive values of the serum/fecal biomarkers for MES 0 in UC patients with normal CRP levels (n = 140).

	AUC	Sensitivity	Specificity	PPV	NPV	Accuracy
LRG	0.62	0.87 (0.78–0.95)	0.41 (0.30–0.52)	0.57 (0.48–0.67)	0.77 (0.64–0.90)	0.63 (0.55–0.71)
FIT	0.72	0.94 (0.88–1.00)	0.42 (0.31–0.54)	0.60 (0.50–0.69)	0.89 (0.78–0.99)	0.67 (0.59–0.75)
Fcal	0.70	0.76 (0.66–0.86)	0.41 (0.30–0.52)	0.54 (0.44–0.64)	0.65 (0.51–0.79)	0.58 (0.50–0.66)

Normal CRP: CRP ≤ 0.2 mg/dL.

*MES* Mayo endoscopic subscore, *UC* ulcerative colitis, *AUC* area under curve, *PPV* positive predictive value, *NPV* negative predictive value, *LRG* leucine-rich alpha-2 glycoprotein, *FIT* fecal immunochemical test, *Fcal* fecal calprotectin, *CRP* C-reactive protein.

Given the above, LRG was considered to be significantly correlated with the endoscopic activity and able to predict MH in UC patients, but the correlation and predictability were not superior to those of fecal markers. The trends were similar in the analysis of patients with normal CRP levels.

### Correlations between serum/fecal biomarkers and histological findings in UC patients

Of the total 166 cases, 158 underwent biopsy for pathological examinations and were evaluated with the Geboes score. Each serum/fecal biomarker was significantly correlated with histologic activity (Spearman’s rank correlation coefficient: LRG: 0.21, *p* = 0.0091, CRP: 0.17, *p* = 0.035, FIT: 0.51, *p* < 0.0001, and Fcal: 0.44, *p* < 0.0001).

The sensitivity, specificity, PPV, NPV, and accuracy of serum/fecal markers in relation to histological remission were calculated with the 158 cases with biopsy. The AUC for histological remission were LRG: 0.65, CRP: 0.61, FIT: 0.74, and Fcal: 0.76, respectively. The sensitivity and specificity for histological remission were LRG: 0.79 and 0.51, CRP: 0.90 and 0.22, FIT: 0.94 and 0.48, and Fcal: 0.81 and 0.50, respectively. Thus, the performance of LRG on histological activity and remission was also not superior to that of fecal markers.

### Correlations between serum/fecal biomarkers and endoscopic findings in CD patients

**Figure 3:**
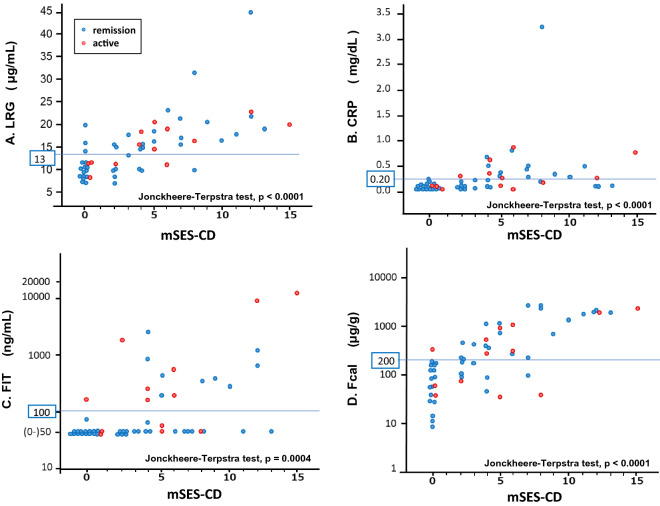
Correlation between the serum/fecal biomarkers and mSES-CD in CD patients. (**A**) LRG, (**B**) CRP, (**C**) FIT, (**D**) Fcal. *mSES-CD* modified simple endoscopic score for Crohn’s disease, *CD* Crohn’s disease, *LRG* leucine-rich alpha-2 glycoprotein, *CRP* C-reactive protein, *FIT* fecal immunochemical test, *Fcal* fecal calprotectin. The red dots indicate the values of patients with active stage and the blue dots indicate the values of patients with remission stage. The specimens with a hemoglobin concentration within this range (0–50 ng/mL) were handled as category, because FIT is not accurate for measuring hemoglobin concentrations of < 50 ng/mL. Parallel blue lines showed the cutoff values of serum/fecal biomarkers in this study (LRG: 13.0 µg/mL, CRP: 0.20 mg/dL, FIT: 100 ng/mL, and Fcal: 200 µg/g).

The correlations between serum/fecal biomarkers and endoscopic findings (mSES-CD) were analyzed. The trend between the serum/fecal markers and the mSES-CD was statistically significant (Jonckheere–Terpstra test: LRG: *p* < 0.0001, CRP: *p* < 0.0001, FIT: *p* = 0.0004, and Fcal: *p* < 0.0001) (Fig. 3).

The sensitivity, specificity, PPV, NPV and accuracy of serum/fecal markers in relation to MH were calculated for the 56 cases. The AUC for complete MH (mSES-CD 0) were LRG: 0.82, CRP: 0.82, FIT: 0.70, and Fcal: 0.88, respectively. The sensitivity and specificity for complete MH were LRG: 0.84 and 0.73, CRP: 0.95 and 0.51, FIT: 0.95 and 0.43, and Fcal: 0.95 and 0.73, respectively (Table 4). Statistical differences were not observed in AUC value between LRG and other markers. The results of sensitivity analysis for MH (mSES-CD 0–2) and active inflammation (mSES-CD > 6) are shown in Supplemental Tables S2 and S3. In addition, the analyses for complete MH and active inflammation using mSES-CD including the strictures score are shown in Supplemental Tables S4 and S5.

**Table 4:** Sensitivity, specificity, and predictive values of the serum/fecal biomarkers for mSES-CD 0 in CD patients.

	AUC	Sensitivity	Specificity	PPV	NPV	Accuracy
LRG	0.82	0.84 (0.68–1.01)	0.73 (0.59–0.87)	0.62 (0.43–0.80)	0.90 (0.79–1.01)	0.77 (0.66–0.88)
CRP	0.82	0.95 (0.85–1.05)	0.51 (0.35–0.67)	0.50 (0.34–0.66)	0.95 (0.85–1.05)	0.66 (0.54–0.78)
FIT	0.70	0.95 (0.85–1.05)	0.43 (0.27–0.59)	0.46 (0.31–0.62)	0.94 (0.83–1.10)	0.61 (0.48–0.74)
Fcal	0.88	0.95 (0.85–1.05)	0.73 (0.59–0.87)	0.64 (0.47–0.82)	0.96 (0.90–1.03)	0.80 (0.70–0.91)

*mSES-CD* modified simple endoscopic score for Crohn’s disease, *CD* Crohn’s disease, *AUC* area under curve, *PPV* positive predictive value, *NPV* negative predictive value, *LRG* leucine-rich alpha-2 glycoprotein, *FIT* fecal immunochemical test, *Fcal* fecal calprotectin, *CRP* C-reactive protein.

We analyzed the association of serum/fecal markers with MH in CD patients with normal serum CRP levels (≤ 0.2 mg/dL). The LRG and Fcal levels were associated with MH in 36 patients with normal serum CRP levels (mSES-CD 0 vs. mSES-CD > 0: LRG *p* = 0.035, FIT *p* = 0.31, and Fcal *p* < 0.0001, respectively) (Supplemental Fig. S2). The AUC for complete MH were 0.71, FIT: 0.57, and Fcal: 0.88, respectively. The sensitivity and specificity for mSES-CD 0 were LRG: 0.83 and 0.50, FIT: 0.94 and 0.22, and Fcal: 0.94 and 0.67, respectively (Table 5). To precisely evaluate the effect of small bowel lesions, the association of serum/fecal markers with MH was analyzed in 20 CD patients who had undergone BAE with normal CRP levels. In this analysis, only Fcal levels were associated with MH in these patients (mSES-CD 0 vs. mSES-CD > 0: LRG 10.1 (7.6–12.2) µg/mL vs. 10.2 (9.0–15.7) µg/mL, *p* = 0.57, FIT 50 (50–50) ng/mL vs. 50 (50–199) ng/mL, *p* = 0.068, and Fcal 45.7 (24.7–157) µg/g vs. 301 (178 – 919) µg/g, *p* = 0.0015, respectively).

**Table 5:** Sensitivity, specificity, and predictive values of the serum/fecal biomarkers for mSES-CD 0 in CD patients with normal CRP levels (n = 36).

	AUC	Sensitivity	Specificity	PPV	NPV	Accuracy
LRG	0.71	0.83 (0.66–1.01)	0.50 (0.27–0.73)	0.63 (0.43–0.82)	0.75 (0.51–1.00)	0.67 (0.51–0.82)
FIT	0.57	0.94 (0.84–1.05)	0.22 (0.03–0.41)	0.55 (0.37–0.72)	0.80 (0.45–1.15)	0.58 (0.42–0.74)
Fcal	0.88	0.94 (0.84–1.05)	0.67 (0.45–0.88)	0.74 (0.56–0.92)	0.92 (0.78–1.07)	0.81 (0.68–0.93)

Normal CRP: CRP ≤ 0.2 mg/dL.

*mSES-CD* modified simple endoscopic score for Crohn’s disease, *CD* Crohn’s disease, *AUC* area under curve, *PPV* positive predictive value, *NPV* negative predictive value, *LRG* leucine-rich alpha-2 glycoprotein, *FIT* fecal immunochemical test, *Fcal* fecal calprotectin, *CRP* C-reactive protein.

Thus, LRG was significantly correlated with the endoscopic activity and could predict MH in CD patients, and the correlation and predictability were equivalent to those of CRP and Fcal. The sensitivity of LRG for MH in CD patients with normal CRP levels was also equivalent to that of Fcal.

Finally, we performed a subanalysis of the correlation between the serum/fecal biomarkers and mSES-CD in CD patients according to the disease location (Supplemental Table S6). Spearman’s rank correlation coefficients between LRG and mSES-CD according to the disease location were L1: 0.57, *p* = 0.033, L2: 0.87, *p* < 0.0001, and L3: 0.69, *p* < 0.0001, respectively. The correlation of LRG appeared to be superior to that of CRP in CD patients with colonic involvement (L2: 0.87, *p* < 0.0001 vs. 0.67, *p* = 0.0066, and L3: 0.69, *p* < 0.0001 vs. 0.44, *p* = 0.021).

## Discussion

In the present study, we evaluated the performance of LRG with regard to the predictability of MH in IBD patients in the clinical setting and compared our findings with those for CRP and two fecal markers. In UC patients, both the correlations with endoscopic activity and predictability of MH of LRG were equivalent to those of CRP but not better than those of fecal markers. In CD patients, both the correlations with endoscopic activity and predictability of MH were equivalent to those of CRP and Fcal.

Two previous studies have evaluated LRG in UC patients. Serada et al.^22^. showed increased levels of LRG in UC patients, and the values were correlated with the clinical activity better than CRP. However, their study lacked an endoscopic evaluation. Shinzaki et al.^23^ demonstrated that LRG correlated with the clinical and colonoscopic activity better than CRP in UC patients, and clinical remission and MH could be discriminated by LRG levels even in patients with normal CRP levels.

Compared with those previous reports, there are several strengths associated with the present study. First, the performance of LRG was compared to the performance of fecal markers as well as that of CRP. Second, evaluations were performed in not only UC but also CD patients. In CD patients in particular, evaluations of the small bowel with BAE were performed if necessary, which is expected to increase the accuracy of the analysis based on the endoscopic findings.

Although we confirmed the correlation of the serum levels of LRG with clinical and endoscopic activity and its predictability of MH in UC, the performance was not superior to that of CRP. The results were inconsistent with those of the previous reports, and the unexpected relatively low correlation and predictability of MH may be attributable to the characteristics of our patients. Our cohort included more patients with lower clinical and endoscopic activity than previous reports. Indeed, approximately 80% of our patients showed no or mild endoscopic activity (MES 0 or 1), while this percentage was 46% (Matts grade 1 or 2) in Shinzaki’s study^23^. Because the sensitivity of CRP for clinical and/or endoscopic activity was relatively low, the difference in performance between LRG and CRP is likely to stand out in cohorts that include more patients with higher activity. The previous reports arbitrarily selected patients with various levels of activity to show the performance of LRG; in contrast, our patients were consecutively recruited and seem more likely to reflect the actual clinical settings. In this context, the clinical usefulness of LRG in the real clinical practice of UC may be more limited than expected.

Although a recent report^36^ showed that no significant difference was observed in LRG levels between UC patients and healthy individuals, some attributes including age, sex, and BMI could affect the values of LRG. In fact, the associations of CRP and Fcal with these attributes have been reported^37–41^. Although the adjustment according to those variables could not be performed due to insufficient data in our analysis, older women may be likely to have higher LRG levels. In fact, of 10 patients (7 UC patients and 3 CD patients) who showed complete MH with an elevated LRG level (> 13 µg/mL) but with normal CRP (≤ 0.20 mg/dL) and normal Fcal (≤ 200 µg/g), 9 were women over 40. Further data collection is required for attributes that affect LRG.

Because cytokines other than IL-6 are also involved in LRG production, the possible influence on the value of LRG under TNFα antagonist was concerned. Hence, we compared AUC value of the serum/fecal biomarkers for complete mucosal healing between IBD patients with and without TNFα antagonist (Supplemental Table S7). In UC patients, AUC of LRG in patients with TNFα antagonist was not superior to that in patients without TNFα antagonist. In CD patients, on the other hand, AUC of LRG in patients with TNFα antagonist was numerically higher than AUC in patients without TNFα antagonist. Because the patient numbers were limited in this regard, additional examinations would be needed in the future.

Few reports have compared the performance of LRG to that of fecal markers based on endoscopic findings. Our results clearly demonstrated that, in UC patients, both fecal markers (Fcal and FIT) were correlated with the endoscopic activity and predicted MH better than LRG. Previous reports have indicated that the correlation with the endoscopic activity and predictability of MH of fecal markers was better than that of CRP^4,7–9^. Because the performance of LRG was not superior to that of CRP in UC patients in the present study, the superiority of fecal markers to LRG is considered reasonable. However, the performance of LRG may also have been underestimated due to the characteristics of our cohort, which included more patients with a lower disease activity than previous studies.

In this study, we examined the performance of LRG in CD patients, and the correlation with the endoscopic activity and predictability of MH of LRG were quite similar to those of CRP and comparable to those of Fcal. The AUCs of both serum markers were higher in CD than in UC. Thus, LRG as well as CRP may be useful for evaluating the disease activity and MH in CD patients, even without fecal tests. In addition, the performance of LRG appears to be superior to that of CRP in CD with colonic involvement. Therefore, LRG might be particularly useful in CD patients with colonic involvement.

In clinical practice of IBD, the evaluation of the disease activity in patients with normal CRP has been challenging. We therefore evaluated the performance of LRG as well as that of fecal markers in UC and CD patients with normal CRP levels. LRG was able to discriminate patients with MH from those with endoscopic activity among both UC and CD patients, as previously shown in UC by Shinzaki et al.^23^. However, the performance of LRG did not surpass that of fecal markers, and in particular, the specificity for MH appeared to be insufficient. Because our cohort included more patients with a lower disease activity, fecal markers may be preferable to LRG to stratify patients with a lower disease activity.

We found no distinct advantage of LRG over CRP or fecal markers in our cohort, which included more patients with a low disease activity than previous studies^22,23^ and largely reflected real clinical practice of IBD. However, serum markers are more convenient to measure for both patients and physicians, and the values of LRG had wider range than those of CRP, suggesting that LRG may be more suitable for evaluating fluctuations in the disease activity than CRP.

The present study is associated with some limitations. First, common drawbacks are the relatively small number of patients and the single-institution design. Second, the effect of concomitant diseases that can potentially increase LRG, such as subtle arthritis, asymptomatic cold, and sinusitis, were not fully evaluated. Third, the small intestine in CD patients was assessed using transanal BAE, and inflammation in the deeper ileum or jejunum might not have been observed. Finally, more patients in the remission stage or with a lower disease activity were included in this study than in previous studies^21,23^, which might have led to results with an insufficient performance of LRG. However, our cohort was consecutively recruited and is expected to largely reflect real clinical practice of IBD.

In conclusion, LRG was correlated with the endoscopic activity and able to predict MH in clinical practice in both UC and CD patients. In UC, however, the performance was not superior to that of fecal markers, whereas in CD, LRG, CRP, and Fcal were equivalently useful. Further experiences and examinations will be required to utilize this new serum marker efficiently in clinical practice of IBD.

## Supplementary Information

Supplementary Information.

## Abbreviations

IBD: Inflammatory bowel disease
UC: Ulcerative colitis
CD: Crohn’s disease
MH: Mucosal healing
CRP: C-reactive protein
IL: Interleukin
Fcal: Fecal calprotectin
FIT: Fecal immunochemical test
LRG: Leucine-rich alpha-2 glycoprotein
BAE: Balloon-associated enteroscopy
CDAI: Crohn’s disease activity index (CDAI)
MES: Mayo endoscopic subscore
SES-CD: Simple endoscopic score for Crohn’s disease
ROC: Receiver operating characteristics curve
AUC: Area under curve
PPV: Positive predictive value
NPV: Negative predictive value
CI: Confidence intervals
