# Supplementary material for: Leucine-rich alpha-2 glycoprotein as a marker of mucosal healing in inflammatory bowel disease
Source: Sci Rep. 2021 May 27;11:11086. doi: 10.1038/s41598-021-90441-x (PMC8160157; doi:10.1038/s41598-021-90441-x)
Supplement: Supplementary file 1 — Supplementary Information. [file 41598_2021_90441_MOESM1_ESM.pdf]

Leucine-rich alpha-2 glycoprotein as a marker of mucosal healing in inflammatory bowel disease

Short title: LRG as biomarker in IBD

Eriko Yasutomi[1*], Toshihiro Inokuchi[1*], Sakiko Hiraoka[1], Kensuke Takei[1], Shoko Igawa[1], Shumpei Yamamoto[1], Masayasu Ohmori[1], Shohei Oka[1], Yasushi Yamasaki[1], Hideaki Kinugasa[1], Masahiro Takahara[1], Keita Harada[1], Masaki Furukawa[2], Kouichi Itoshima[2], Ken Okada[2], Fumio Otsuka[2,3], Takehiro Tanaka[4], Toshiharu Mitsuhashi[5], Jun Kato[6], Hiroyuki Okada[1]

1. Department of Gastroenterology and Hepatology, Okayama University Graduate School of Medicine, Dentistry and Pharmaceutical Sciences, Okayama, Japan

2. Department of Laboratory Medicine, Okayama University Hospital, Okayama, Japan.

3. Department of General Medicine, Okayama University Graduate School of Medicine, Dentistry and Pharmaceutical Sciences, Okayama, Japan

4. Department of Pathology, Okayama University Graduate School of Medicine, Dentistry and Pharmaceutical Sciences, Okayama, Japan

5. Center for Innovative Clinical Medicine, Okayama University Hospital, Okayama, Japan.

6. Department of Gastroenterology, Graduate School of Medicine, Chiba University, Chiba, Japan

*E Yasutomi, and T Inokuchi contributed equally to this work.

Supplemental table 1 Sensitivity, specificity, and predictive values of the serum/fecal biomarkers for MES 2 or 3 in UC patients.

	AUC	Sensitivity	Specificity	PPV	NPV	Accuracy
LRG	0.61	0.50 (0.33-0.67)	0.68 (0.60-0.76)	0.27 (0.16-0.38)	0.85 (0.78-0.92)	0.64 (0.57-0.72)
CRP	0.55	0.22 (0.08-0.36)	0.85 (0.79-0.91)	0.26 (0.09-0.42)	0.82 (0.76-0.88)	0.73 (0.66-0.80)
FIT	0.85	0.78 (0.64-0.92)	0.84 (0.78-0.90)	0.54 (0.40-0.69)	0.94 (0.90-0.98)	0.83 (0.77-0.89)
Fcal	0.77	0.66 (0.49-0.82)	0.71 (0.63-0.79)	0.35 (0.23-0.47)	0.90 (0.84-0.95)	0.70 (0.63-0.77)

MES, Mayo endoscopic subscore; UC, ulcerative colitis; AUC, area under curve; PPV, positive predictive value; NPV, negative predictive value; LRG, leucine-rich alpha-2 glycoprotein; CRP, C-reactive protein; FIT, fecal immunochemical test; Fcal, fecal calprotectin.

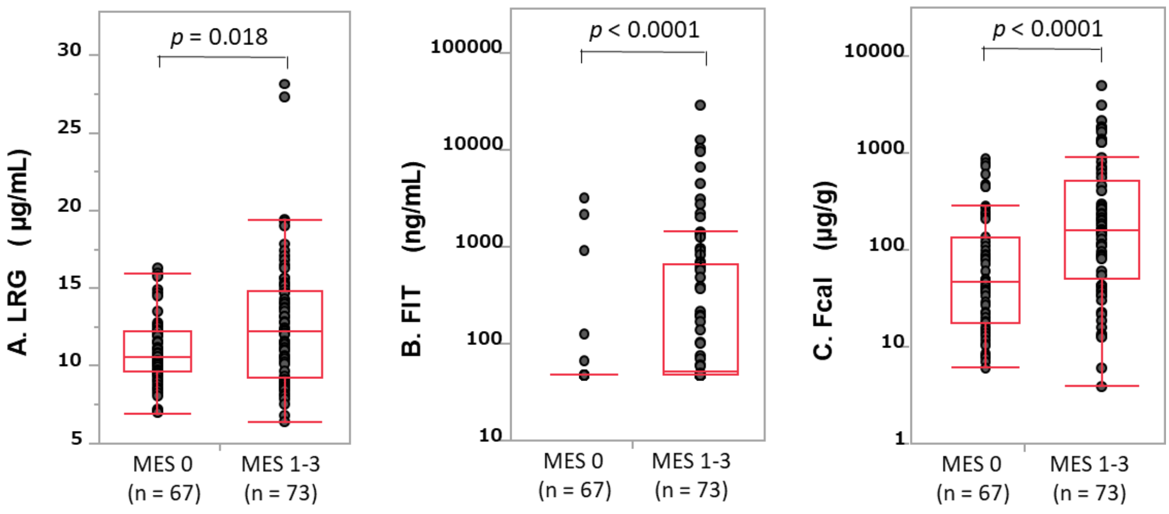

Supplemental figure 1. Correlation between MES and the serum/fecal biomarkers in UC patients with normal CRP* (n = 140)

*normal CRP, CRP ≤ 0.2 mg/dL

Supplemental table 2. Sensitivity, specificity, and predictive values of the serum/fecal biomarkers for mSES-CD 0-2 in CD patients.

	AUC	Sensitivity	Specificity	PPV	NPV	Accuracy
LRG	0.89	0.81 (0.66-0.96)	0.83 (0.70-0.97)	0.81 (0.66-0.96)	0.83 (0.70-0.97)	0.82 (0.72-0.92)
CRP	0.85	0.92 (0.82-1.03)	0.60 (0.42-0.78)	0.67 (0.51-0.82)	0.90 (0.77-1.03)	0.75 (0.64-0.86)
FIT	0.73	0.92 (0.82-1.03)	0.50 (0.32-0.68)	0.62 (0.46-0.77)	0.88 (0.73-1.04)	0.70 (0.58-0.82)
Fcal	0.88	0.85 (0.71-0.98)	0.80 (0.66-0.94)	0.79 (0.63-0.94)	0.86 (0.72-0.99)	0.82 (0.72-0.92)

mSES-CD, modified simple endoscopic score for Crohn's disease; CD, Crohn's disease; AUC, area under curve; PPV, positive predictive value; NPV, negative predictive value; LRG, leucine-rich alpha-2 glycoprotein; CRP, C-reactive protein; FIT, fecal immunochemical test; Fcal, fecal calprotectin.

Supplemental table 3. Sensitivity, specificity, and predictive values of the serum/fecal biomarkers for mSES-CD > 6 in CD patients.

	AUC	Sensitivity	Specificity	PPV	NPV	Accuracy
LRG	0.87	0.93 (0.79-1.06)	0.60 (0.45-0.74)	0.43 (0.26-0.61)	0.96 (0.89-1.04)	0.68 (0.56-0.80)
CRP	0.75	0.64 (0.39-0.89)	0.74 (0.61-0.87)	0.45 (0.23-0.67)	0.86 (0.75-0.97)	0.71 (0.60-0.83)
FIT	0.63	0.50 (0.24-0.76)	0.76 (0.63-0.89)	0.41 (0.18-0.65)	0.82 (0.70-0.94)	0.70 (0.58-0.82)
Fcal	0.87	0.86 (0.67-1.04)	0.62 (0.47-0.77)	0.43 (0.25-0.61)	0.93 (0.83-1.02)	0.68 (0.56-0.80)

mSES-CD, modified simple endoscopic score for Crohn’s disease; CD, Crohn’s disease; AUC, area under curve; PPV, positive predictive value; NPV, negative predictive value; LRG, leucine-rich alpha-2 glycoprotein; CRP, C-reactive protein; FIT, fecal immunochemical test; Fcal, fecal calprotectin.

Supplemental table 4. Sensitivity, specificity, and predictive values of the serum/fecal biomarkers for mSES-CD (including strictures score) 0 in CD patients.

	AUC	Sensitivity	Specificity	PPV	NPV	Accuracy
LRG	0.82	0.83 (0.66-1.01)	0.71 (0.57-0.85)	0.58 (0.39-0.77)	0.90 (0.79-1.01)	0.75 (0.64-0.86)
CRP	0.80	0.94 (0.84-1.05)	0.50 (0.34-0.66)	0.47 (0.31-0.64)	0.95 (0.85-1.05)	0.64 (0.52-0.77)
FIT	0.69	0.94 (0.84-1.05)	0.42 (0.26-0.58)	0.44 (0.28-0.59)	0.94 (0.83-1.05)	0.59 (0.46-0.72)
Fcal	0.88	0.94 (0.84-1.05)	0.71 (0.57-0.85)	0.61 (0.43-0.79)	0.96 (0.90-1.03)	0.79 (0.68-0.89)

mSES-CD, modified simple endoscopic score for Crohn's disease; CD, Crohn's disease; AUC, area under curve; PPV, positive predictive value; NPV, negative predictive value; LRG, leucine-rich alpha-2 glycoprotein; CRP, C-reactive protein; FIT, fecal immunochemical test; Fcal, fecal calprotectin.

Supplemental table 5. Sensitivity, specificity, and predictive values of the serum/fecal biomarkers for mSES-CD (including strictures score) > 6 in CD patients.

	AUC	Sensitivity	Specificity	PPV	NPV	Accuracy
LRG	0.88	0.94 (0.83-1.05)	0.64 (0.49-0.79)	0.53 (0.35-0.71)	0.96 (0.89-1.04)	0.73 (0.62-0.85)
CRP	0.81	0.71 (0.49-0.92)	0.79 (0.67-0.92)	0.60 (0.39-0.81)	0.86 (0.75-0.97)	0.77 (0.66-0.88)
FIT	0.67	0.53 (0.29-0.77)	0.79 (0.67-0.92)	0.53 (0.29-0.77)	0.79 (0.67-0.92)	0.71 (0.60-0.83)
Fcal	0.82	0.82 (0.64-1.00)	0.64 (0.49-0.79)	0.50 (0.31-0.69)	0.89 (0.78-1.01)	0.70 (0.58-0.82)

mSES-CD, modified simple endoscopic score for Crohn’s disease; CD, Crohn’s disease; AUC, area under curve; PPV, positive predictive value; NPV, negative predictive value; LRG, leucine-rich alpha-2 glycoprotein; CRP, C-reactive protein; FIT, fecal immunochemical test; Fcal, fecal calprotectin.

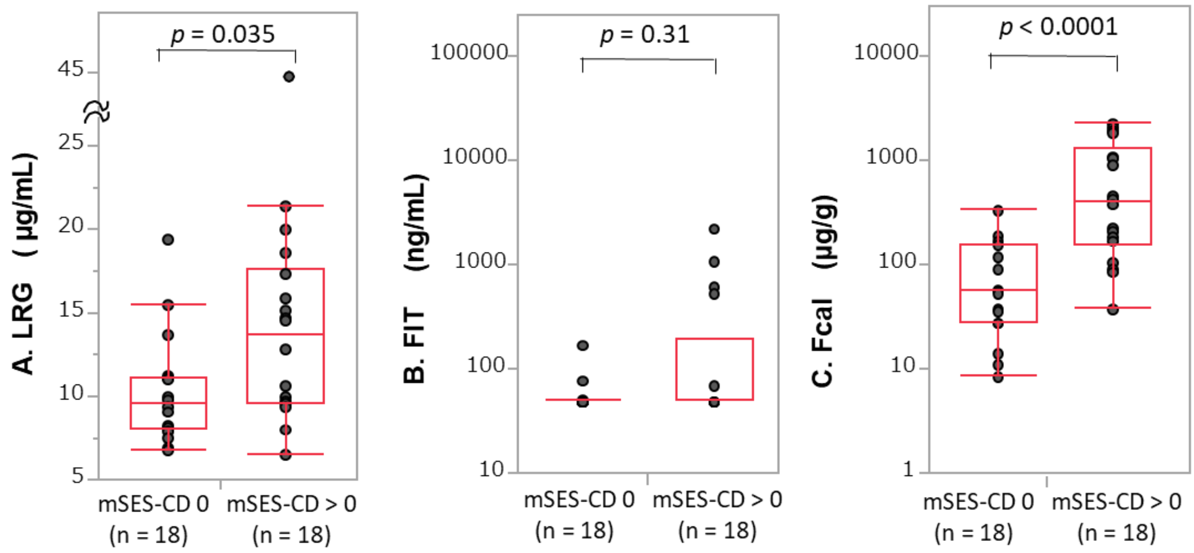

Supplemental figure 2. Correlation between mSES-CD and the serum/fecal biomarkers in CD patients with normal CRP* (n = 36)

*normal CRP, CRP ≤ 0.2 mg/dL

Supplemental table 6. Correlation between the serum/fecal biomarkers and mSES-CD in CD patients according to the disease location.

	L1: ileal (n = 14)		L2: colonic (n = 15)		L3: ileocolonic (n = 27)	
	r	*p*	*r*	*p*	*r*	*p*
LRG	0.57	0.033	0.87	<0.0001	0.69	< 0.0001
CRP	0.72	0.004	0.67	0.0066	0.44	0.021
FIT	0.62	0.018	0.53	0.044	0.38	0.052
Fcal	0.48	0.086	0.78	0.0007	0.72	< 0.0001

mSES-CD, modified simple endoscopic score for Crohn’s disease; CD, Crohn’s disease; LRG, leucine-rich alpha-2 glycoprotein; CRP, C-reactive protein; FIT, fecal immunochemical test; Fcal, fecal calprotectin.

Supplemental table 7. Comparisons of AUC value of the serum/fecal biomarkers for complete mucosal healing between IBD patients with and without TNFα antagonist.

	Ulcerative colitis			Crohn's disease		
	Total	TNFα antagonist		Total	TNFα antagonist	
		with	without		with	without
	n = 166	n = 21	n = 145	n = 66	n = 33	n = 23
LRG	0.61	0.40	0.64	0.82	0.82	0.75
CRP	0.59	0.68	0.58	0.82	0.82	0.72
FIT	0.75	0.60	0.77	0.70	0.62	0.79
Fcal	0.72	0.68	0.71	0.88	0.94	0.70
